# Paradoxical hepcidin–iron dysregulation in primary sclerosing cholangitis

**DOI:** 10.1371/journal.pone.0361385

**Published:** 2026-10-27

**Authors:** Petr Kordac, Milan Hajek, Monika Cahova, Mojmir Hlavaty, Petr Sedivy, Monika Dezortova, Dita Pajuelo, Kamila Balusikova, Jan Brezina, Pavel Drastich

**Affiliations:** 1 Department of Diagnostic and Interventional Radiology, Institute for Clinical and Experimental Medicine, Magnetic Resonance unit, Prague, Czech Republic; 2 Department of Physiology, Faculty of Science, Charles University, Prague, Czech Republic; 3 Centre for Experimental Medicine, Institute for Clinical and Experimental Medicine, Prague, Czech Republic; 4 Department of Hepatogastroenterology, Institute for Clinical and Experimental Medicine, Prague, Czech Republic; 5 Department of Biochemistry, Cell and Molecular Biology, 3rd Faculty of Medicine, Charles University, Prague, Czech Republic; Lady Davis Institute for Medical Research, CANADA

## Abstract

**Background:**

Primary sclerosing cholangitis (PSC) is a chronic, progressive liver disease characterised by biliary inflammation. Its exact aetiology remains unclear. This study aimed to use a combination of diagnostic methods and blood analysis to better understand the metabolic changes accompanying PSC.

**Methods:**

PSC patients (n = 60) and healthy controls (n = 60) were evaluated by magnetic resonance imaging and spectroscopy (hepatic fat and iron content), ultrasonography, standard blood analysis expanded with markers of iron metabolism, and colonoscopy.

**Results:**

PSC patients exhibited significant liver fibrosis and elevated liver enzymes. Serum hepcidin was increased (*p* < 0.01), but PSC patients did not show any markers of anaemia. Serum iron concentration and transferrin saturation were increased compared with healthy controls (*p* < 0.0001 and *p* < 0.001, respectively) but did not reach levels seen in hemochromatosis. In contrast, the liver iron content was lower in PSC patients (*p* < 0.0001).

**Conclusion:**

PSC patients demonstrated an atypical pattern of iron metabolism: elevated circulating iron and depleted liver iron stores despite increased hepcidin levels. This paradox suggests a disruption of hepcidin-mediated iron regulation, potentially sustaining a proinflammatory environment. Further investigation is warranted to clarify underlying mechanisms and therapeutic implications.

**Trial Registration:**

Clinical Trials I.D.: NCT06026449

## 1. Introduction

Primary sclerosing cholangitis (PSC) is a rare, chronic, and progressive autoimmune disease characterised by biliary inflammation, leading to bile duct strictures, fibrosis, liver cirrhosis, and eventually to end-stage liver disease. Its prevalence varies considerably between regions, ranging from 0 to 32 cases per 100 000 individuals worldwide, and is typically diagnosed in middle-aged men. Patients may be asymptomatic or experience jaundice, fever, pruritus, abdominal pain, fatigue [1–3]. Despite medical treatment, the disease progresses and liver transplantation is currently the only therapeutic option significantly improving the overall survival of patients with more advanced disease [4]. Approximately 70–80% of patients have developed concomitant inflammatory bowel disease (IBD), with ulcerative colitis being the most common [5,6].

The exact pathogenesis of PSC remains poorly understood, but a genetic predisposition, immune dysregulation, bile acid toxicity and intestinal dysbiosis likely play important roles [7]. The natural course of PSC shows high variability and the prognosis is difficult to predict, with an unmet need for reliable prognostic biomarkers for this disease.

Disturbances of iron homeostasis are increasingly recognised as important contributors to the pathogenesis and progression of chronic liver diseases. While hereditary hemochromatosis represents the classical example of iron-driven liver injury [8], dysregulated iron metabolism has also been described in several other hepatic disorders. In metabolic dysfunction-associated steatotic liver disease, hyperferritinaemia has been associated with an increased risk of liver related outcomes and more advanced liver fibrosis in non-invasive testing [9]. Similarly, in alcohol-related liver disease, hepatic iron overload has been linked with higher risk of liver related outcomes and hepatocellular carcinoma [10].

In PSC, however, very little is known about iron metabolism. The situation is more complex with the concomitant IBD, which is frequently associated with iron deficiency and anaemia [11–13]. Additionally, chronic inflammation itself can disturb iron metabolism [14,15]. Systemic iron levels are tightly regulated, primarily through intestinal absorption by the action of hepcidin, an acute-phase hormone and key regulator of iron metabolism, that limits iron absorption and release from stores (Fig 1) [16–20]. The primary source of hepcidin is the liver, but it is also expressed in the kidney and heart, and may also be present in bile [18–21].

**Fig 1:**
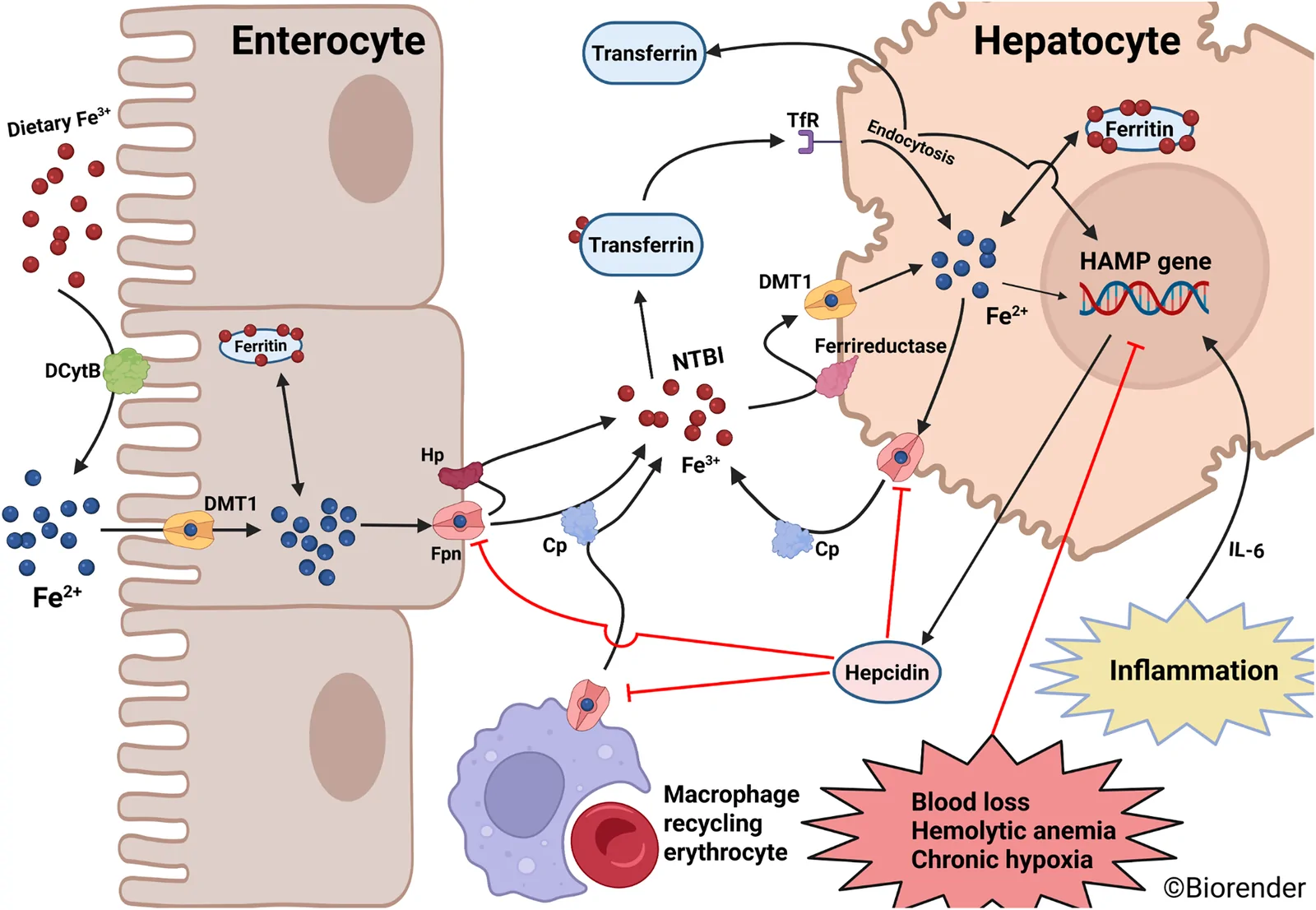
Iron absorption and metabolism and the hepcidin regulatory pathway. Dietary inorganic iron must be reduced by brush border ferrireductase (duodenal cytochrome B) prior to absorption by enterocytes. Iron is then transported across the basolateral membrane by ferroportin, where it is oxidised either by plasmatic ceruloplasmin or the transmembrane protein hephaestin. Once in the bloodstream, iron binds primarily to transferrin and is taken up by hepatocytes by receptor-mediated endocytosis. When necessary, iron is released from hepatocytes via ferroportin. Hepcidin tightly regulates the level of iron in the blood by preventing release of iron via the degradation or blockade of ferroportin, and by inhibiting iron absorption from the intestine, through proteasome degradation of divalent metal transporter 1. Cp – ceruloplasmin, DCytB – duodenal cytochrome B, DMT1 – divalent metal transporter 1, Fpn – ferroportin, Hp – hephaestin, IL-6 – interleukin 6, NTBI – non-transferrin bound iron, TfR – transferrin receptor.

The diagnosis of PSC is based on a combination of clinical symptoms, laboratory tests, and imaging methods. Of the imaging methods, magnetic resonance (MR) techniques, have significant roles in the diagnosis of PSC, monitoring the course of treatment, and in predicting outcomes [1,6,22]. In addition, ^1^H MR spectroscopy can be used to quantify hepatic fat [23,24]. Finally, MR can be used to calculate R2 and R2* relaxation rates, which provide information about liver iron status [24–26].

This study aimed to comprehensively characterise PSC patients with an emphasis on iron metabolism using a multimodal diagnostic method.

## 2. Methods

### 2.1. Study participants

This cross-sectional single-centre study included sixty patients with PSC (Age = 40 ± 11 years, BMI = 25.3 ± 4.6 kg/m^2^), who underwent MR imaging and spectroscopy, ultrasonography, and blood analysis. PSC was diagnosed based on current guidelines [27]. The exclusion criteria included coeliac disease, liver transplantation, decompensated liver cirrhosis with a Model for End-Stage Liver Disease score of ˃ 15, recurrent bacterial cholangitis, and having MR-incompatible implants or a pacemaker. Patients with concomitant IBD treated with biologic or other innovative treatments were also excluded.

The control group consisted of sixty healthy individuals (Age = 40 ± 10 years, BMI = 25.5 ± 3.4 kg/m^2^) matched for sex, age and BMI to the PSC group. The study was conducted in compliance with the ethical principles of the Declaration of Helsinki and with the approval of the local ethics committee. Participant recruitment took place between 7.2.2023 and 2.12.2025, and all provided written informed consent before study inclusion.

### 2.2. Blood analysis

The biochemical analyses were performed in a certified clinical laboratory following standard diagnostic protocols. Results were compared with local hospital laboratory physiological ranges. Hepcidin assays were performed in serum samples that were stored at −80°C. Mature bioactive hepcidin was measured by hepcidin-25 (bioactive) high-sensitivity ELISA kits (EIA-5782, DRG International, USA).

### 2.3. Ultrasonography examination

Ultrasonography examinations were performed with an Aixplorer MACH 30 system (SuperSonic Imagine, Aix-en-Provence, France) equipped with a 3.5 MHz abdominal curved transducer (SC6–1). Two-dimensional shear wave elastography was used to measure liver stiffness. The elastography measurements were performed by experienced operators following the international recommendations [28]. A cut-off for exclusion of liver fibrosis was set at stage 7.1 kPa, based on previously published data [28–30].

### 2.4. Colonoscopy

At study entry, all potentially eligible PSC patients underwent colonoscopy after standard recommended preparation with low-volume polyethylene glycol solution with simethicone following the international recommendation [31] under adequate sedation. If present, the severity of associated ulcerative colitis was evaluated using the Mayo Endoscopic score (MES).

### 2.5. Magnetic resonance spectroscopy and imaging

MR examinations were performed with a 3T MR system VIDA (MAGNETOM Vida, Siemens, Germany) equipped with a 30-channel surface coil and a 32-channel spine coil. The examination protocol included the HASTE (half-Fourier acquisition single-shot turbo spin-echo) localizer in three orthogonal planes and standard Siemens LiverLab [32] protocol including volumetric interpolated breath-hold examination (VIBE) e-Dixon and q-Dixon sequences in the transversal plane for liver volumetry (VIBE e-Dixon: TR = 3.97 ms, TE = 1.29 ms, resolution 1.2 × 1.2 × 3 mm, flip angle = 9°, 80 slices; acceleration factor 2 × 2 caipirinha). Moreover, single voxel spectroscopic sequence HISTO [33] (STEAM sequence; TR = 3000 ms; 5 spectra during one breath hold with TEs = 12, 24, 36, 48, and 72 ms) was placed in the volume of interest (VOI). The VOI (40 × 30 × 25 mm) and VIBE region of interest were placed in the liver segment V/VIII in an area without visible big vessels. All sequences were measured in the supine position during a single breath-hold. Automatic and manual shimming were combined to reach a half-width below 50 Hz. The MR examination took 30–40 minutes to perform.

Hepatic fat content (HFC) was calculated from fat fraction values assessed by a HISTO sequence using a correction described by Longo et al. [34]. HFC > 5.43% is considered as advanced liver steatosis [23]. To estimate liver iron content (LIC), mean R2 and R2* relaxation rates of water were calculated for both groups as reciprocal values of the T2 and T2* relaxation times of water obtained by HISTO and VIBE q-Dixon sequences.

### 2.6. Data analysis

Data were analysed with GraphPad Prism 10.6 (GraphPad Software, USA). Results were reported as means ± SD or medians (Q1–Q3). Normality was assessed with the D’Agostino–Pearson test. Between-group differences were evaluated using the unpaired *t*-test or Mann-Whitney U test. The relationship of variables was assessed by Pearson or Spearman correlation analysis. Multigroup comparisons were performed with one-way ANOVA and Tukey’s post hoc or Kruskal-Wallis tests. A *p*-value < 0.05 was considered significant.

## 3. Results

### 3.1. Blood analysis

Bilirubin, aspartate aminotransferase, alanine aminotransferase, alkaline phosphatase (ALP), and gamma-glutamyl transferase (GGT) were elevated in the PSC patients (all *p* < 0.0001; Table 1). Albumin was lower than in the control group (p < 0.0001), but within normal physiological range. C-reactive protein median value was 3.6 (1.6–10.2) mg/l in the PSC patients and 0.8 (0.4–2.8) mg/l in the control group. Serum iron, total iron binding capacity (TIBC), transferrin, and transferrin saturation were higher in the PSC group. After adjusting for sex, the difference in iron level remained significant in men (*p* < 0.001) but not in women (*p* = 0.323). The iron level exceeded the normal physiological upper limit of 28.6 µmol/l for men and 25.9 µmol/l for women in 41% of the PSC patients. TIBC was above the normal limit of 72 µmol/l in 55% of the PSC patients. Differences in other measured blood parameters in the PSC and control groups were not significant and the values were within their normal physiological ranges (Table 1). The serum bioactive hepcidin level was higher in the PSC patients than in the healthy control group (*p* < 0.01). After adjusting for sex, the difference remained significant in men (*p* < 0.01) but not in women.

**Table 1:** Basic characteristic of primary sclerotic cholangitis (PSC) patients and healthy controls. Body mass index (BMI), total iron binding capacity (TIBC), mean erythrocytes volume (MCV), mean erythrocytes haemoglobin weight (MCH), mean haemoglobin concentration in erythrocytes (MCHC), C-reactive protein (CRP), aspartate aminotransferase (AST), alanine aminotransferase (ALT), alkaline phosphatase (ALP), gamma-glutamyl transferase (GGT). All values are shown as average ± SD.

Parameters	Controls	PSC patients	*p*-value
**Men/women**	n = 46/14	n = 46/14	
**Age [years]**	40 ± 10	40 ± 11	ns
**BMI [kg/m** ^**2**^ **]**	25.5 ± 3.4	25.3 ± 4.6	ns
**Iron [µmol/l]**	18.6 ± 7.2	26.8 ± 12.0	< 0.0001
**Men**	18.5 ± 7.0	27.3 ± 10.7	< 0.0001
**Women**	18.8 ± 8.1	25.4 ± 15.1	ns
**TIBC [µmol/l]**	66.9 ± 9.5	77.0 ± 14.3	< 0.0001
**Hepcidin [ng/ml]**	16.0 ± 10.4	24.1 ± 15.6	< 0.01
**Men**	18.5 ± 10.9	27.4 ± 14.8	< 0.01
**Women**	10.1 ± 5.7	13.0 ± 12.7	ns
**Ferritin [µg/l]**	161 ± 112	208 ± 157	ns
**Transferrin [g/l]**	2.7 ± 0.4	3.1 ± 0.6	< 0.0001
**Men**	2.6 ± 0.4	3.0 ± 0.6	< 0.001
**Women**	2.7 ± 0.3	3.2 ± 0.4	< 0.01
**Transferrin sat. [%]**	28.4 ± 12.0	35.1 ± 16.0	< 0.01
**Erythrocytes [10** ^**12**^ **/l]**	4.94 ± 0.4	4.94 ± 0.5	ns
**Haemoglobin [g/l]**	149 ± 10	148 ± 17	ns
**Haematocrit [l/l]**	0.431 ± 0.028	0.431 ± 0.044	ns
**MCV [fl]**	87.8 ± 4.6	87.5 ± 5.1	ns
**MCH [pg]**	30.2 ± 1.8	30.0 ± 2.1	ns
**MCHC [g/l]**	345 ± 9	343 ± 10	ns
**Albumin [g/l]**	49.3 ± 2.6	46.2 ± 4.3	< 0.0001
**CRP [mg/l]**	1.9 ± 2.9	8.0 ± 11.1	< 0.0001
**Bilirubin [µmol/l]**	13.5 ± 10.7	32.1 ± 24.8	< 0.0001
**AST [µkat/l]**	0.5 ± 0.3	1.3 ± 0.8	< 0.0001
**ALT [µkat/l]**	0.6 ± 0.1	1.7 ± 1.1	< 0.0001
**ALP [µkat/l]**	1.3 ± 0.3	6.2 ± 3.6	< 0.0001
**GGT [µkat/l]**	0.5 ± 2.3	6.6 ± 6.2	< 0.0001

Anti-inflammatory medication was reported in 46 out of the 60 PSC patients. Comparison of treated and untreated patients showed no significant differences in any measured laboratory parameter. None of the participants reported current oral iron supplementation or previous intravenous iron therapy. In uni- and multivariable regression analyses using serum hepcidin as the dependent variable, serum ferritin was the only independent predictor of hepcidin concentration (See S1 Data).

### 3.2. Ultrasonography examination

In 13 of the PSC patients (22%) ultrasonography revealed a deformed liver shape characterised by disproportion in the size of the left and right lobes. Hypertrophy of the caudate lobe was observed in 9 patients (15%), surface nodularity was observed in 6 (10%), a coarse, heterogeneous echotexture was observed in 21 (39%), an increased echogenicity was seen in 16 (27%), and 8 (14%) patients had already developed liver cirrhosis. The liver stiffness was 12.4 ± 12.7 kPa, vena portae diameter was 10.7 ± 2.2 mm and venous flow was 19.7 ± 3.9 cm/s.

The liver stiffness measurement was ≤ 7.1 kPa in 23 PSC patients (F0-1) and > 7.1 kPa in 36 (F2+). The ultrasonography examination was missing in 1 patient entirely.

In the control group, the liver stiffness was 5.3 ± 1.0 kPa, the vena portae diameter was 10.3 ± 4.2 mm and venous flow was 21.4 ± 3.8 cm/s. The liver stiffness measurement was ≤ 7.1 kPa in 57 individuals (F0-1) and > 7.1 kPa in 3 individuals.

### 3.3. Colonoscopy

All PSC patients enrolled in the study underwent total colonoscopy except for one patient with incomplete colonoscopy due to a large inguinal hernia. At the time of study, 9 of the 59 patients were not diagnosed with IBD, which was subsequently confirmed endoscopically and histologically. One patient was not evaluated because he had previously undergone a subtotal colectomy. All patients with concomitant PSC and IBD had ulcerative colitis with a maximal documented extent of pancolitis (extensive disease based on Montreal classification [35]). Endoscopic disease activity was assessed at the study-entry colonoscopy. Endoscopic remission (MES 0) was confirmed in 25 of the 49 patients, mild disease (MES 1) in 20, and 4 patients were considered to have moderately active colonic disease (MES 2). MES was not correlated with serum iron, hepcidin, or ferritin.

### 3.4. Magnetic resonance spectroscopy and imaging

Both the R2 and R2* relaxation rates of water were lower in PSC patients than in healthy controls (*p* < 0.0001 for both), which indicated a lower LIC in the PSC group. However, both the R2 and R2* relaxation rates are also reduced owing to liver fibrosis [36]. To distinguish the involvement of fibrosis, we divided the PSC patients into two groups by their elastography values: without liver fibrosis (F0-1; liver stiffness ≤ 7.1 kPa) and with liver fibrosis (F2+ ; liver stiffness > 7.1 kPa; Fig 2, Table 2). From controls, the 3 individuals with liver fibrosis were excluded. The R2 relaxation rate remained higher in controls (38.6 ± 4.5 s^-1^) than in F0-1 (33.9 ± 2.9 s^-1^) and F2+ patients (30.3 ± 4.7 s^-1^; *p* < 0.001). The R2* relaxation rate was also higher in controls (45.4 ± 10.4 s^-1^) than in F0-1 (40.3 ± 5.8 s^-1^) and F2+ patients (35.8 ± 7.6 s^-1^; *p* < 0.001).

**Table 2:** Basic characteristic of primary sclerotic cholangitis (PSC) patients and healthy controls measured by magnetic resonance and ultrasonography. For selected parameters, the PSC group was further divided into patients without fibrosis (F0-1, liver stiffness ≤ 7.1 kPa) and with fibrosis (F2+ , liver stiffness > 7.1 kPa). HFC – hepatic fat content. All values are shown as average ± SD.

Parameters	Controls	PSC patients		*p*-value
**Liver stiffness [kPa]**	5.3 ± 1.0	12.4 ± 12.7		< 0.0001
**Vena portae diameter [mm]**	10.3 ± 4.2	10.7 ± 2.2		ns
**Venous flow [cm/s]**	21.4 ± 3.8	19.7 ± 3.9		ns
**HFC [%]**	2.8 ± 2.7	3.0 ± 4.5		ns
**R2 relaxation rate of water [s** ^**-1**^ **]**	38.7 ± 4.4	31.8 ± 4.5		< 0.0001
R2* relaxation rate of water[s ^**-1**^ **]**	44.9 ± 10.3	37.7 ± 7.3		< 0.0001
	(n = 57)	**F0-1** (n = 23)	**F2+** (n = 36)	
**Liver stiffness [kPa]**	5.2 ± 0.8	5.8 ± 0.6^☥☥☥^	16.5 ± 14.7^***^	< 0.0001
**HFC [%]**	2.9 ± 2.7	4.3 ± 5.8	1.7 ± 1.9	ns
**R2 relaxation rate of water [s** ^**-1**^ **]**	38.6 ± 4.5	33.9 ± 2.9^***,☥☥^	30.3 ± 4.7^***^	< 0.001
R2* relaxation rate of water [s ^**-1**^ **]**	45.4 ± 10.4	40.3 ± 5.8^☥^	35.8 ± 7.6^***^	< 0.0001

** = p < 0.01 vs controls; *** = p < 0.001 vs controls, ☥ = p < 0.05 vs F2+ ; ☥☥☥ = p < 0.001 vs F2+

**Fig 2:**
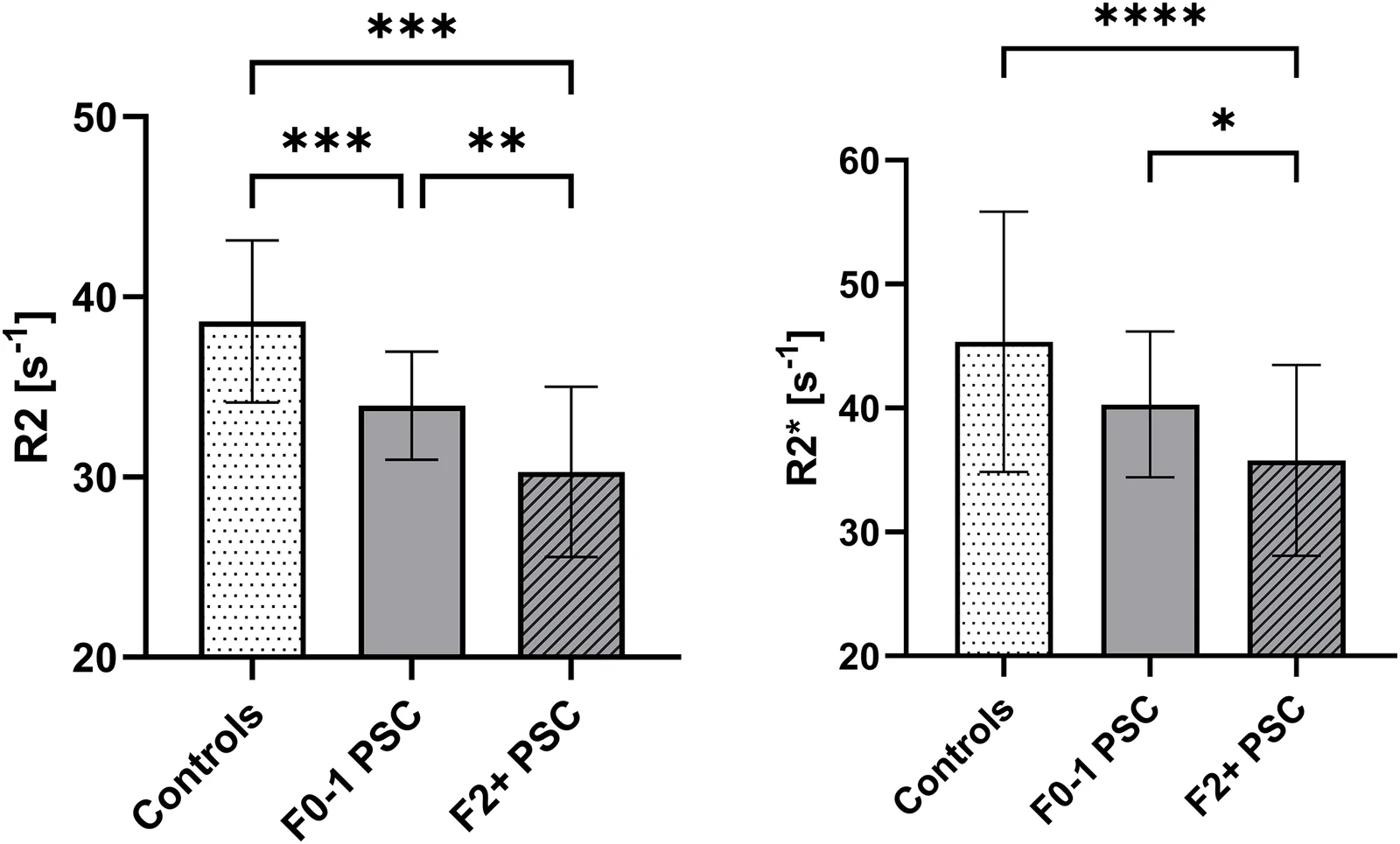
R2 and R2* relaxation rates of water in healthy controls (liver stiffness ≤ 7.1 kPa; n = 57), primary sclerosing cholangitis (PSC) patients without liver fibrosis (F0-1, liver stiffness ≤ 7.1 kPa; n = 23) and PSC patients with fibrosis (F2+, liver stiffness > 7.1 kPa; n = 36). * = p < 0.05; ** = p < 0.01; *** = p < 0.001.

Both R2 and R2* relaxation rates of water correlated with liver stiffness (Fig 3). HFC of PSC patients did not differ from that of healthy controls. Advanced liver steatosis was identified in seven PSC patients and ten controls.

**Fig 3:**
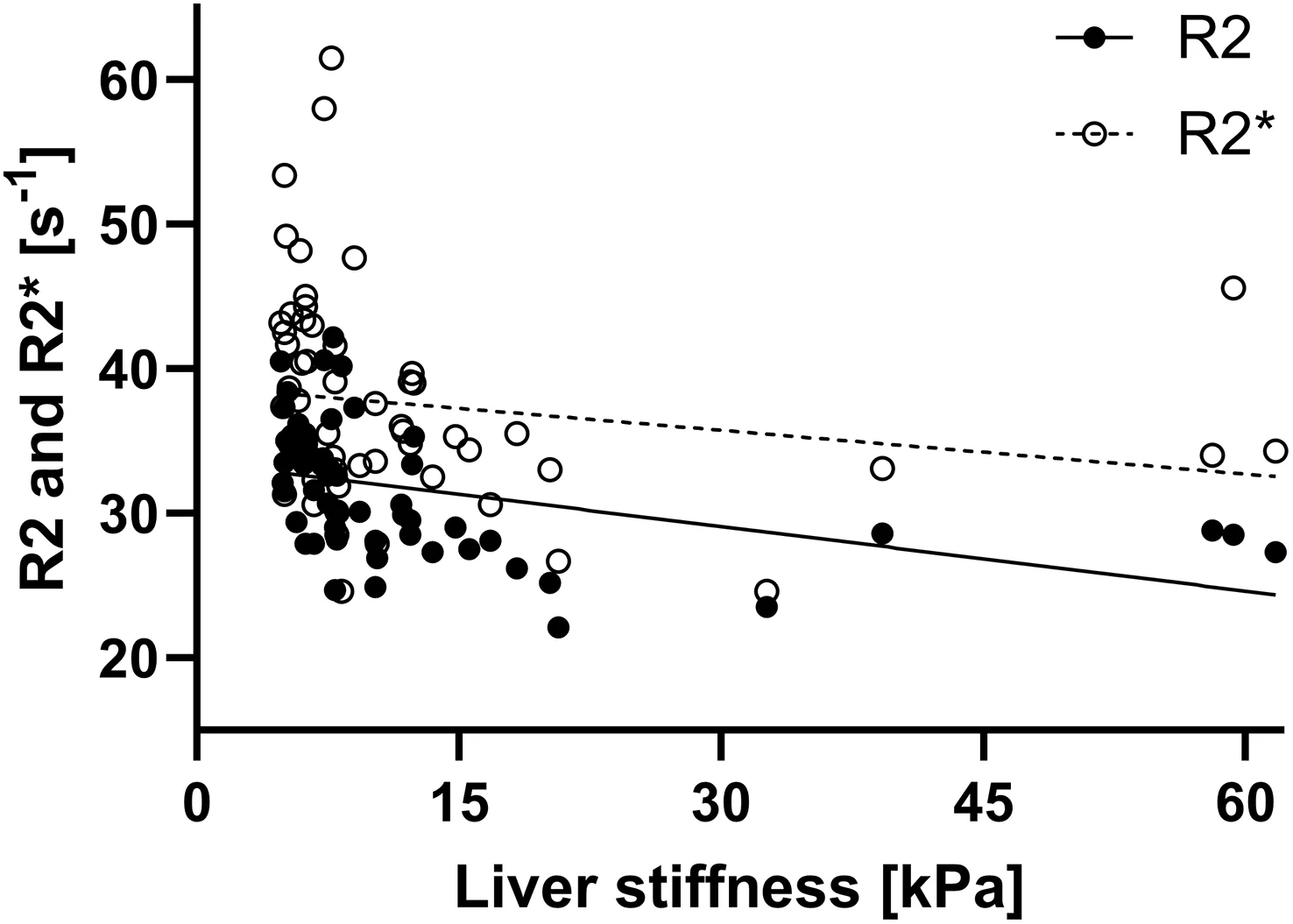
Spearman correlation between liver stiffness and both R2 (r = −0.617 and p < 0.0001) and R2* (r = −0.406 and p < 0.001) relaxation rates of water in primary sclerosing cholangitis patients (n = 59).

## 4. Discussion

In this study, we characterised a rather substantial sample of PSC patients using a combination of imaging methods and blood analyses, with particular attention to iron metabolism. MR and ultrasonography confirmed a wide range of morphological variations in PSC patients that were caused by changes in the bile ducts and varying degrees of fibrosis, which is common for PSC [1,2]. Liver cirrhosis was observed in 8 (14%) PSC patients. Morphological variations were also present in the gallbladder and spleen.

As expected, PSC cohort exhibited significantly higher liver enzymes, when compared to healthy controls, with predominant elevation of ALP and GGT. All patients in the PSC cohort were either in the early stage of disease, fibrosis or compensated advanced chronic liver disease, with preserved liver function. Serum iron concentrations were elevated in PSC patients, especially among men. This was unexpected given the co-occurrence of IBD in 85% of our PSC patients, as iron concentration is typically reduced in such cases [12,13]. Although elevated iron normally stimulates hepcidin production, increased hepcidin would be expected to reduce circulating iron concentrations through inhibition of iron export and absorption [19]. Therefore, the simultaneous elevation of both serum iron and hepcidin observed in our cohort suggests altered iron homeostasis.

Hepcidin levels typically increase during chronic inflammation leading to decreased plasma iron while tissue iron stores remain unchanged [19]. As both PSC and IBD are inflammatory diseases, we anticipated this pattern. Consistent with this assumption, CRP concentrations were significantly higher in PSC patients than in healthy controls, confirming greater systemic inflammatory activity in the PSC cohort. Although systemic inflammation was significantly greater in PSC patients, CRP was not independently associated with serum hepcidin concentration in multivariable analysis. Nevertheless, despite elevated hepcidin levels, PSC patients demonstrated increased serum iron together with reduced liver iron content. Instead, we observed low tissue iron and elevated plasma iron concentrations despite significantly increased hepcidin levels in our PSC patients. Importantly, the simultaneous elevation of serum iron and hepcidin should not occur if hepcidin-mediated regulatory pathway works properly. Plasma iron originates from three sources: the diet, liver storage, recycled erythrocytes. All these pathways are directly regulated by hepcidin, which suppresses them by inhibiting Fpn and/or DMT1 [16,19,21]. Our results indicate that erythrocytes are not a source of increased plasma iron, as erythrocyte count and their parameters, haematocrit, and haemoglobin were within the normal limits.

The R2 relaxometry results suggest that the increased plasma iron likely originated from liver storage. Hepcidin negatively regulates both the efflux and influx of iron in hepatocytes. In hepatocytes, influx is mediated by both DMT1 and transferrin receptor [17]. Brasse-Lagnel, et al. [20] reported that DMT1 was inhibited at lower hepcidin concentrations in enterocytes *ex vivo* before Fpn was affected. If this pathway also occurs in hepatocytes, it would help explain why plasma iron remained in the blood rather than returning to liver stores. However, we still lack an explanation for the increase in plasma iron levels. PSC is a progressive disease with hepatocyte damage [37], which may eventually result in iron leakage and contribute to the increased circulating iron pool. The increased serum hepcidin concentration may be the consequence of the ongoing inflammatory status.

On the other hand, opposing regulatory mechanism might also be involved. Reduced tissue iron levels, potentially a consequence of tissue damage, might lead to elevated plasma iron level, subsequently stimulating hepcidin expression. This effect may be more pronounced in men, as women are protected by regular bleeding, which could potentially explain why significant increases in serum iron and hepcidin were observed only in men. Co-occurrence of IBD was probably not the cause of elevated hepcidin or serum iron concentrations in the PSC patients, as no correlation was found.

When placed in the context of existing literature, our results appear distinct. In a study of autoimmune liver diseases (PBC/PSC; n = 34, with only 4 PSC cases), serum hepcidin concentrations were significantly lower than in healthy controls [38]. Moreover, a recent meta-analysis found that hepcidin levels tend to be lower in hepatitis C and alcoholic liver disease, and higher in non-alcoholic fatty liver disease, while data on PSC were insufficient for firm conclusions [39]. In contrast, our cohort of 60 PSC patients demonstrated significantly elevated serum hepcidin accompanied by increased serum iron and transferrin saturation but reduced hepatic iron stores. This paradoxical combination has not been described in larger PSC cohorts to date and may indicate a unique disturbance of iron homeostasis in PSC.

### 4.1. Study limitations

Information on menopausal status, contraception, and menstrual cycle phase in female participants were lacking. R2* relaxometry did not confirm reduced hepatic iron in the F0–1 PSC subgroup. However, R2 mapping was more appropriate for assessing iron deficiency whereas R2* relaxometry is more suitable for iron excess [40].

## 5. Conclusion

Our study reveals novel insights into PSC, highlighting unexpected abnormalities of iron metabolism. We found increased serum iron, transferrin saturation, and hepcidin, suggesting a disruption in iron homeostasis. Concurrently, ^1^H MR spectroscopy showed reduced liver iron content. While differences in other measured modalities between PSC patients and controls were insignificant, these findings are particularly noteworthy. Most hepatocellular and cholestatic diseases are classically associated with hepatic iron overload rather than deficiency — making our observation of reduced hepatic iron alongside elevated serum iron in PSC a novel and unusual finding. Although the direct clinical impact of these results may be limited at present, the primary aim of this experimental study was to bring novel insights into the pathogenesis of PSC. As iron metabolism in PSC remains poorly understood, further research is warranted to fully understand these findings and their implications for PSC treatment, potentially leading to future changes in clinical practice.

## Supporting information

S1 Data Supplementary data. (DOCX)

## Abbreviation:

ALP: Alkaline phosphatase
DMT1: Divalent metal transporter
Fpn: Ferroportin
GGT: Gamma-glutamyl transferase
HFC: Hepatic fat content
IBD: Inflammatory bowel disease
LIC: Liver iron content
MES: Mayo Endoscopic score
MR: Magnetic resonance
PSC: Primary sclerosing cholangitis
TE: Echo time
TIBC: Total iron binding capacity
TR: Repetition time
VIBE: Volumetric interpolated breath-hold examination
VOI: Volume of interest
