# Supplementary material for: Paradoxical hepcidin–iron dysregulation in primary sclerosing cholangitis
Source: PLoS One. 2026 Oct 27;21(10):e0361385. doi: 10.1371/journal.pone.0361385 (PMC13712708; doi:10.1371/journal.pone.0361385)
Supplement: Supplementary file 1 [file pone.0361385.s001.docx]

# **Supplementary data**

Univariable and multivariable linear regression analyses were performed to evaluate factors associated with serum hepcidin concentration in PSC patients (Table S1) and control group (Table S2). Hepcidin was used as the dependent variable. Ferritin, haemoglobin, CRP, transferrin saturation, and sex were selected as predictors based on their biological and clinical relevance to hepcidin regulation and iron homeostasis. All parameters, but sex, were log-transformed. Each predictor was first assessed separately in univariable analysis, followed by a multivariable model including all five predictors simultaneously.

*Table S1 Univariable and multivariable linear regression analyses of factors associated with serum hepcidin in PSC patients*

| **Predictor** | **Univariable β** | **p** | **Adjusted R²** | **Multivariable β** | **p** |
| --- | --- | --- | --- | --- | --- |
| **log(Ferritin)** | 0.835 | <0.0001 | 0.626 | 0.773 | <0.0001 |
| **log(Haemoglobin)** | 4.528 | <0.0001 | 0.289 | 1.639 | 0.053 |
| **log(CRP)** | −0.011 | 0.921 | −0.017 | −0.023 | 0.751 |
| **log(Transferrin sat.)** | 0.686 | 0.001 | 0.168 | 0.032 | 0.827 |
| **Sex** | -0.960* | 0.0016 |  | ±0.089^†^ | 0.433 |

*β denotes the unstandardized regression coefficient. Univariable analyses included 60 patients, expect for transferrin saturation (n = 56). The multivariable analysis included 56 patients with complete data.*

**For sex in the univariable analysis, the value represents the mean difference in log-transformed hepcidin between women and men (women minus men: −0.960; 95 % CI −1.540 to −0.381; pooled two sample t-test, p = 0.0016).*

*†Sex was included as a categorical variable in the multivariable model (men = -0.089; women = 0.089).*

**Multivariable equation:** *Log(Hepcidin) = -9.268 + 0.772 log(Ferritin) + 1.639 log(Haemoglobin) - 0.023 log(CRP) + 0.0324 log(Transferrin sat.) + sex offset (-0.089 for men; +0.089 for women)*

*Table S2 Univariable and multivariable linear regression analyses of factors associated with serum hepcidin in control group*

| **Predictor** | **Univariable β** | **p** | **Adjusted R²** | **Multivariable β** | **p** |
| --- | --- | --- | --- | --- | --- |
| **log(Ferritin)** | 0.849 | <0.0001 | 0.676 | 0.891 | <0.0001 |
| **log(Haemoglobin)** | 4.478 | 0.016 | 0.120 | -0.258 | 0.868 |
| **log(CRP)** | 0.211 | 0.042 | 0.081 | 0.056 | 0.440 |
| **log(Transferrin sat.)** | -0.022 | 0.933 | -0.026 | -0.033 | 0.844 |
| **Sex** | -0.622* | 0.0298 |  | ±0.088^†^ | 0.437 |

*β denotes the unstandardized regression coefficient. Univariable and multivariable analysis included 40 controls with complete data set.*

**For sex in the univariable analysis, the value represents the mean difference in log-transformed hepcidin between women and men (women minus men: −0.622; 95 % CI −1.176 to −0.067; Welch two sample t-test, p = 0.0298).*

*†Sex was included as a categorical variable in the multivariable model (men = -0.088; women = 0.088).*

**Multivariable equation:** *Log(Hepcidin) = -0.334 + 0.891 log(Ferritin) - 0.258 log(Haemoglobin) + 0.056 log(CRP) - 0.033 log(Transferrin sat.) + sex offset (-0.088 for men; +0.088 for women)*

*Conclusion*

In univariable analyses of PSC patients, ferritin, haemoglobin, transferrin saturation, and sex were significantly associated with hepcidin, whereas CRP was not. After simultaneous adjustment for all five predictors, ferritin remained the only statistically significant independent predictor of hepcidin (β = 0.773, p < 0.0001). Haemoglobin did not reach statistical significance in the multivariable model (β = 1.639, p = 0.053), while no independent associations were observed for CRP, transferrin saturation, or sex. The final multivariable model explained 69.6 % of the variance in log-transformed hepcidin (R² = 0.696; adjusted R² = 0.666).

In controls, ferritin was strongly associated with log-hepcidin in univariable regression (β = 0.849, p < 0.0001). Haemoglobin and CRP were also significant in univariable analyses, but neither remained significant after adjustment. In the multivariable model, only ferritin remained significantly associated with log-hepcidin (β = 0.891, p < 0.0001). The final multivariable model explained 70.0 % of the variance in log-transformed hepcidin (R² = 0.700; adjusted R² = 0.656).
